# Overdiagnosis in Lung Cancer Screening: Still the Unavoidable Cost of Doing Business?

**DOI:** 10.1016/j.atssr.2025.01.002

**Published:** 2025-01-29

**Authors:** Riccardo Orlandi, Luigi Rolli, Ugo Pastorino

**Affiliations:** 1 Department of Thoracic Surgery, University of Milan, Milan, Italy; 2 Department of Thoracic Surgery, IRCCS Istituto Nazionale dei Tumori Foundation, Milan, Italy

Lung cancer (LC) remains one of the most formidable challenges in public health and claims more lives globally than any other form of cancer.^1^ Late diagnosis plays a pivotal role in determining its dismal survival rates. In an effort to improve outcomes, LC screening (LCS) programs that are based on low-dose computed-tomography (LDCT) scanning have been introduced.^2^ The rationale is clear: early detection could lead to early intervention, potentially reducing mortality rates.^3^

However, the introduction of LCS has brought with it a complex and often underappreciated issue—overdiagnosis. As screening technologies advance and become more prevalent, the risk of overdiagnosis in LCS is becoming an increasingly pressing concern.^4^

Here we explore the implications of overdiagnosis in LCS, by drawing on evidence from clinical studies and examining the ethical, psychological, and economic consequences. We also consider strategies to mitigate this issue and discuss the need for a balanced approach that maximizes the benefits of early detection while minimizing harm.

## Understanding Overdiagnosis

*Overdiagnosis* refers to the detection of real cancer that, if left undetected, would not have caused symptoms or death during a patient's lifetime.^5^ This concept is often misunderstood or conflated with related issues such as false positive results, where a patient incorrectly receives a diagnosis of cancer that does not exist.

Three conditions are required for defining overdiagnosis in cancer screening^6^: a reservoir of slow-growing or nongrowing cancer, the capability to detect this reservoir, eligible screening participants with a significant risk of mortality from causes unrelated to cancer. Therefore, overdiagnosis can occur in 2 ways during LCS: first, a patient with progressive, screen-detected cancer may die of other causes before the cancer progresses to the point of generating symptoms; second, some screen-detected cancers may not be progressive and would never advance to a stage where they cause symptoms. In both situations, it is almost impossible to determine whether an individual screen-detected case represents overdiagnosis. Indeed, currently, clear diagnostic criteria for overdiagnosis are still lacking, and the rate of overdiagnosis can be estimated only at a population level rather than on an individual level, on the basis of 2 main approaches^7^: the early-stage vs late-stage approach and the cumulative incidence. The latter is the most commonly used method for estimating overdiagnosis, by comparing the cumulative incidence of cancer in a screened population with the cumulative incidence in an unscreened population. The difference in incidence between these 2 groups represents the number of overdiagnosed cases. The former is based on the concept that screening leads to an increase in the detection of early-stage cancers: if this rise in early-stage cancers is not offset by a corresponding decrease in symptomatic (reflected by late-stage) cancers, the difference represents the number of overdiagnosed cases.

Although most thoracic experts still believe that LC should always be treated, evidence that indolent LCs exist is increasing. Indeed, long-term observational studies have revealed that some pure ground-glass opacities without any solid component may represent premalignant or preinvasive diseases.^8^, ^9^, ^10^, ^11^ These conditions may warrant surveillance for several years without any detrimental effect on survival because of their indolent behavior. Such indolent tumors are unlikely to progress into deadly cancers within 10 years. Even when these tumors are resected years after diagnosis, patients demonstrate excellent long-term survival, with 10-year overall survival rates approaching 100%.^8^^,^^12^ Few studies even reported prolonged survival in some patients with pure lepidic adenocarcinomas without therapy.^13^^,^^14^

The challenge lies in distinguishing between these types of tumors at the time of detection, a distinction that is often difficult with current screening modalities.^15^ Indeed, the tumor size plays a pivotal role in determining the chance of advanced disease, but it is not enough alone to predict the risk accurately. For adenocarcinomas with a maximum diameter of less than 20 mm, the rate of N+ staging is approximately 14%, without nodal involvement observed in tumors smaller than 10 mm. In contrast, for tumors measuring between 20 and 30 mm, the rate of N+ staging doubles to 30%.^16^

To date, neither a definitive guideline nor a clear understanding on the biologic behavior of LC allows us to forecast which preinvasive tumor will aggressively progress and which will not. Therefore, the precautionary principle compels us to adopt a proactive approach even in patients with evidence of a suspected or confirmed preinvasive tumor, such as atypical adenomatous hyperplasia, adenocarcinoma in situ, or minimally invasive adenocarcinoma. Nonetheless, this approach could lead to overtreatment and harm.

## Evidence of Overdiagnosis

In randomized controlled trials of LDCT-based LCS, the overall estimate of overdiagnosis is 29%,^17^ ranging from 12.9% to 67.2%. The variation in overdiagnosis rates across different trials can be partially attributed to differences in screening protocols, population demographics, and follow-up periods.

A key challenge in assessing overdiagnosis is the lack of long-term follow-up in many studies. Moreover, variations in overdiagnosis rates across different populations suggest that certain groups may be more susceptible to overdiagnosis than others. Another critical point is the concept of contamination, which refers to the unintended screening of individuals in the control group during or after the active screening phase of a study. This phenomenon can occur when members of the control group independently seek screening or are inadvertently screened as part of routine medical care. Contamination undermines the distinction between the screened and unscreened groups and leads to an underestimation of overdiagnosis. By blurring the lines between the groups, contamination diminishes the ability to assess the true benefits and risks of the screening intervention accurately. This issue, in turn, complicates the evaluation of screening efficacy in reducing mortality or morbidity. Several factors contribute to overdiagnosis in LCS:

1. Advances in imaging technology: The sensitivity of imaging techniques, particularly LDCT, has dramatically increased, enabling the detection of very small lung nodules that may progress slowly, without ever becoming symptomatic in a patient’s lifespan.
2. Increased screening coverage: As screening becomes more widely available and recommended for broader populations, the likelihood of detecting indolent tumors will increase.
3. Lowering thresholds for biopsy and diagnosis: With more aggressive screening protocols, the tendency is to biopsy and diagnose smaller nodules, some of which may represent overdiagnosed cases.

## Consequences of Overdiagnosis

The consequences of overdiagnosis in LCS are multifaceted, affecting patients, health care systems, and public perceptions of screening programs. One of the most immediate consequences of overdiagnosis is the physical harm caused by unnecessary treatments. Patients with a diagnosis of indolent tumors may undergo surgery, radiation, or other locoregional treatments, such as cryoablation, microwave ablation, or radiofrequency ablation, which may carry significant risks and side effects. For patients whose tumors would not have progressed, these treatments represent harm without benefit.

The psychological impact of overdiagnosis is also profound. Having a diagnosis of cancer, even if the cancer is indolent, can lead to significant anxiety, stress, and decreased quality of life. The label of “cancer patient” carries with it a burden of fear and uncertainty, which can persist even after treatment.

Overdiagnosis also has substantial economic implications. The costs associated with unnecessary treatments, follow-up procedures, and long-term surveillance can strain health care resources. In addition to direct medical costs, there are indirect costs, such as lost productivity resulting from illness or treatment. In resource-limited settings, the allocation of funds toward overdiagnosed cases may divert resources away from patients who need more urgent care, thus exacerbating health disparities.

Eventually, overdiagnosis can erode public trust in LCS programs. As awareness of overdiagnosis grows, individuals may become more skeptical of the benefits of screening, and this could lead to decreased participation rates. This skepticism can be compounded by the difficulty of communicating the concept of overdiagnosis to the public, whose fear of cancer is pervasive.

## Ethical Considerations

Medical ethics is grounded in the principle of *primum non nocere*. Overdiagnosis challenges this tenet by introducing the possibility that well-intentioned interventions could lead to unnecessary suffering. The ethical balance lies in ensuring that the benefits of screening—reduced mortality and morbidity—outweigh the harms of overdiagnosis and overtreatment.

A critical aspect of addressing the ethical implications of overdiagnosis is ensuring that patients are fully informed about the risks and benefits of screening. Informed consent must go beyond simply obtaining a signature; it requires meaningful communication that allows patients to understand the potential outcomes of screening, including the possibility of overdiagnosis. In Western countries, patients are required to undergo a shared decision-making process to be enrolled in LCS programs. However, because patients are typically focused on the favorable aspects of screening, the physician has a duty to highlight the other side of the coin, including the risk of overdiagnosis, to raise awareness of its potential consequences.

## Mitigating Overdiagnosis

Several strategies are currently being explored to reduce overdiagnosis in LCS:

1. Risk-based screening approaches: Rather than applying a 1-size-fits-all screening protocol, risk-based approaches tailor screening recommendations on the basis of an individual’s risk factors, such as age, smoking history, and family history.
2. Personalized screening programs: Advances in genetic and molecular profiling may enable the development of personalized screening programs that take into account an individual’s unique tumor biology, thereby allowing for more targeted and effective screening.
3. Refining criteria for biopsy and diagnosis: Establishing more stringent criteria for invasive procedures can help to reduce the number of indolent tumors that are detected and treated. Using a higher threshold for nodule size or growth rate, as well as focusing on solid component and volumetric expansion, could help to distinguish between aggressive and indolent tumors.
4. Advances in imaging techniques: New imaging technologies and techniques are being developed to better differentiate between aggressive and indolent tumors: radiomics, deep learning, and machine learning techniques are likely the future of LCS.
5. Integration of functional imaging: Accurate use of fluorine-18 fluorodeoxyglucose positron emission tomography scanning could be crucial in defining biologic aggressiveness and in predicting the risk of invasiveness of LCs.
6. Shared multidisciplinary decisions: Regular multidisciplinary meetings that include at least a thoracic surgeon, a nuclear medicine physician, a thoracic radiologist, a medical oncologist, and a respiratory physician can effectively support the active surveillance of suspicious nodules.
7. Long-term studies: Long-term follow-up studies are needed to better understand the natural history of tumors detected through screening and to refine estimates of overdiagnosis.
8. Public health initiatives: Educating the public about the risks and benefits of screening, including the potential for overdiagnosis, is vital for ensuring informed participation in screening programs.

In conclusion, overdiagnosis in LCS is a significant and complex issue that has far-reaching implications for patients, health care systems, and society as a whole. Although screening offers the promise of early detection and improved outcomes, it also carries the risk of detecting cancers that may never have caused harm, thus leading to unnecessary treatments and emotional distress. To address the challenges of overdiagnosis, it is essential to strike a balance between the benefits of early detection and the potential harms of overtreatment. This balance requires ongoing research, innovation in screening techniques, and a commitment to patient-centered care that prioritizes informed decision making and shared understanding. As we move forward, it will be crucial to evaluate and adapt LCS practices to the best interests of patients and society continually. By doing so, we can work toward a future where LCS fulfills its potential to save lives without causing unnecessary harm.
